# Evolution and adoption of contributor role ontologies and taxonomies

**DOI:** 10.1002/leap.1496

**Published:** 2022-09-20

**Authors:** Mohammad Hosseini, Julien Colomb, Alex O. Holcombe, Barbara Kern, Nicole A. Vasilevsky, Kristi L. Holmes

**Affiliations:** 1 Department of Preventive Medicine, Northwestern University Feinberg School of Medicine, Chicago, Illinois, USA; 2 Institute of Biology, Humboldt-Universität Zu Berlin, Berlin, Germany; 3 School of Psychology, University of Sydney, Sydney, Australia; 4 The John Crerar Library, University of Chicago, Chicago, Illinois, USA; 5 Oregon Clinical & Translational Research Institute, Oregon Health & Science University, Portland, Oregon, USA; 6 Galter Health Sciences Library and Learning Center, Northwestern University Feinberg School of Medicine, Chicago, Illinois, USA

**Keywords:** contributor roles, contributorship, authorship, ethical attribution, research integrity

## Abstract

Contributor Role Ontologies and Taxonomies (CROTs) are standard vocabularies to describe individual contributions to a scholarly project or research output. Contributor Roles Taxonomy (CRediT) is one of the most widely used CROTs, and has been adopted by numerous journals to describe author’s contributions, and recently formalized as a ANSI/NISO standard. Despite these developments, there is still much work left to be done to improve how CROTs are used across different research domains, research output types, and scholarly workflows. In this paper, we describe how CROTs could be extended to include roles from various disciplines in an ethical and inclusive manner. We explore potential approaches to apply CROTs to diverse research objects and various disciplines; as well as envision their integration into various scholarly workflows, such as promotion and tenure in academic institutions. Lastly, we discuss potential mechanisms for wide adoption and use. While acknowledging that improving current systems of attribution is a slow and iterative process, we believe that engaging the community in the evolution of CROTs will ultimately enhance the ethical attribution of credit and responsibilities in scholarly publications.

## INTRODUCTION

Most science today is conducted by teams, which are sometimes dispersed across institutions and geographical locations. Not only do researchers in teams *deserve* credit for their contributions, they also *need* credit to demonstrate their knowledge and skills for hiring, promotion and funding applications (Resnik, 2005; Shapiro et al., 1994). Additionally, the public and the scientific community should be able to decipher who is accountable for different aspects of a research project. For example, it should be clear who to approach to understand more about specific parts of the research process, especially when questions are raised about errors or possible fraudulent practices (Hosseini & Gordijn, 2020).

Although useful in naming (some) contributors to research projects, conventional authorship bylines do not entail a description of tasks conducted by each author. This shortcoming was the impetus for using contributorship statements, which describe *who did what* using free text (Rennie et al., 2008). While containing more information than authorship bylines, free-text contributorship statements yield an inconsistent description of similar tasks across publications, and complicate summarizing/tallying researchers’ contributions across projects and publications. Furthermore, these statements are ‘not a usable piece of metadata associated with a specific research output, not searchable, and not surfaced by indexers’ (Allen et al., 2019), so contributorship statements do not comply with Findable, Accessible, Interoperable, Reusable (FAIR) principles (FORCE11, n.d.). Accordingly, in fields such as biomedical sciences where researchers make diverse contributions to dozens of publications each year and are involved in different publications to different extents, contributorship statements do not allow developing a reliable and consistent summary of individual contributions.

Previous papers have discussed ways that Contributor Role Ontologies and Taxonomies (CROTs; i.e., formal vocabularies that specify individual contributions to research projects and outputs) address some of these issues by facilitating a more consistent attribution of credit and responsibilities while adhering to FAIR principles (e.g., Allen et al., 2014; Allen et al., 2019; Holcombe, 2019). As computational and machine-readable descriptions of contributions, CROTs not only enhance the attribution of credit, they also catalyse ‘the necessary cultural shift to evolve scholarship to grow towards open knowledge infrastructures’ (Vasilevsky et al., 2020). The quick uptake of CROTs by numerous publishers (e.g., Wiley, Sage, Elsevier, Springer) and repositories (e.g., DARIAH-DE, Zenodo), and also CRediT’s recent standardization by the National Information Standards Organization (NISO) as well as its approval by the American National Standards Institute (ANSI; NISO, 2022a, 2022b) highlight CROTs’ success and the significant role they can play in scholarly publications. On that basis, strategizing about their future development is essential.

In this article, we provide a window to some challenges and possibilities involved in further development of CRediT and other CROTs. Specifically, we will discuss:

- Extension of CROTs in an ethical manner;
- Application of CROTs to various research objects and disciplines;
- Integration of CROTs into different scholarly workflows; and
- Supporting CROTs for adoption and use.

While there might be additional issues surrounding the future of CROTs, this paper focuses on the above, and will *not* address challenges of using CROTs in parallel with authorship guidelines, for example the requirement of many journals that every author should contribute to the writing of a manuscript. This is a somewhat separate issue that we do not have space to address, although it is related to the larger discussion about the future of CROTs (see more in Holcombe, 2019 and Hosseini et al., 2022a).

## ETHICAL EXTENSION OF ROLES

Scientific work is diverse—there is no single scientific method, and the activities of different fields vary enormously (Hepburn & Andersen, 2021). In addition, necessary contribution types in different fields change over time. For example, 25 years ago, there were essentially no genome-wide association studies, but after the first was published in 2002 they rapidly became common (Ozaki et al., 2002), and necessitated new methods and contribution types (e.g., RNA-sequencing, exome sequencing). Since science and scientific methods evolve regularly, CROTs too should evolve to reflect new contribution types. So far, among major CROTs, only Contributor Role Ontology (CRO, https://github.com/data2health/contributor-role-ontology) and Taxonomy of Digital Research Activities in the Humanities (TaDiRAH, https://vocabs.dariah.eu/tadirah/en/) have been revised after the release of their first version.

Since the research community is the primary beneficiary group of CROTs, the process of extending them requires meaningful engagement with the community. Our vision for this process to be ethical and inclusive involves three steps: (I) Identification of candidate roles; (II) Deciding what roles to include/exclude in CROTs’ standard list; and (III) Integrating new roles to the existing list of roles. Here, we explore possible approaches to these steps and mention involved challenges.

### Identification of candidate roles

One can argue that CROTs cannot (and should not) add *just any role* to their list or else the list of roles will become longer than necessary and discourage further adoption and use. Therefore, compiling a shortlist of candidate roles with clear and straightforward descriptions is prudent, and can be achieved using different approaches. Using community feedback is one way to create such a shortlist. Currently, different CROTs have developed mechanisms to solicit feedback from the community. For instance, CRO and TaDiRAH are open to public user feedback via the issue tracker on their GitHub page. Under CASRAI management, CRediT had an online community forum and organized several annual user meetings, and the newly appointed NISO committee will establish a community interest group to remain engaged with the community. Exploring direct community feedback can help standing committees in compiling a shortlist of clearly defined candidate roles. However, using this approach is not always easy because not every member of the community would engage with GitHub or interest groups, and more importantly, different users might provide contradictory suggestions.

**FIGURE 1:**
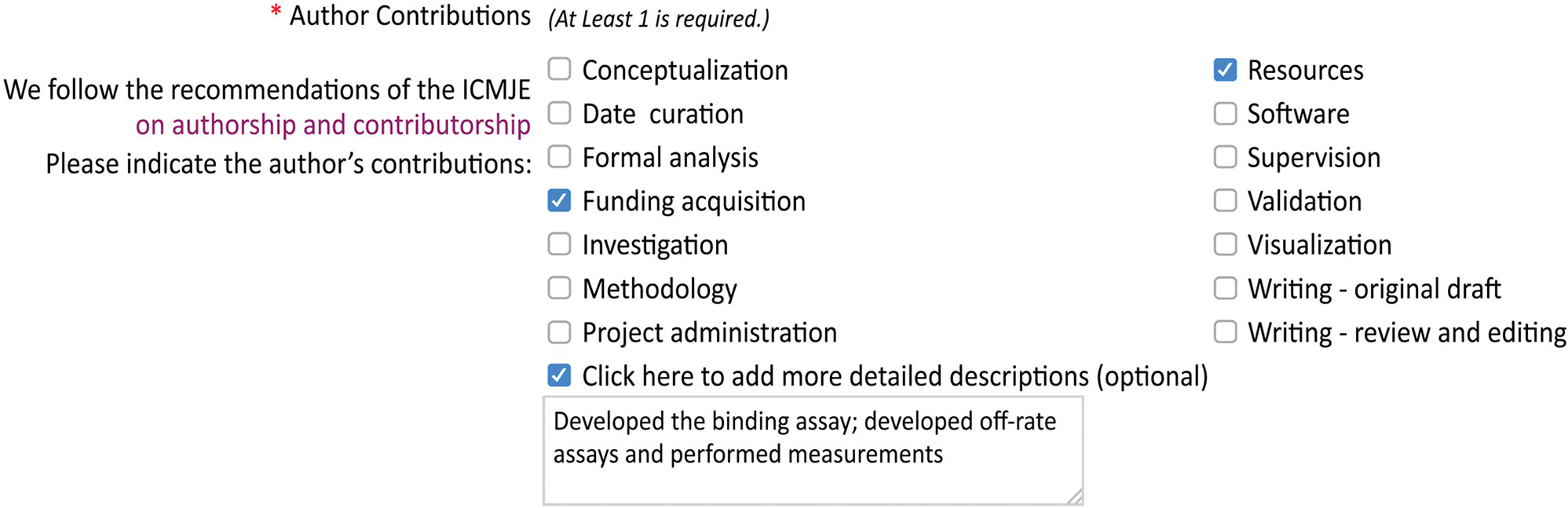
The optional free-text section provided by the journal *eLife*. *Source*: https://elifesciences.org/inside-elife/f39cfcf5/enabling-the-contributor-roles-taxonomy-for-author-contributions.

Candidate roles can also be inferred from acknowledgement/contributorship statements. This approach was adopted by CRediT developers in compiling the initial list of roles, as described in the document reporting on the IWCSA Workshop: ‘a text analysis of the ‘Acknowledgements’ section of a large sample of scientific publications was performed’ (Allen et al., 2012, p. 16). A more refined way of employing this method would entail analysing acknowledgement/contributorship statements of journals that have adopted one of the CROTs. For instance, while using CRediT, the journal *eLife* (eLife, 2017) also provides the option to add more detailed descriptions about contributions (Fig. 1). Perhaps if the contributions of all involved individuals were accurately captured by CRediT roles, the optional box to add more information would remain unused. However, in cases where this text box is used, analysing detailed descriptions with text-mining techniques could result in inferring new candidate roles. Some challenges of adopting this approach pertains to the role that human judgement might play in adding extra information and an inconsistent reflection of contributions. While one hopes that roles that cannot be adequately described using CROTs’ existing list of roles are always mentioned in the acknowledgement sections or in the optional text box provided by the journal, in the absence of a clear structure or guideline, these roles might be omitted or added in a haphazard manner. Some groups (or the corresponding author) might decide against adding extra information and/or use the closest term from the existing list of roles to specify the missing roles. For instance, the text box shown in Fig. 1 notes the development of ‘binding assays’ and ‘off-rate assays’ in the free-text section. Some groups might assume these roles are covered by CRediT’s role of Investigation and proceed without using the optional text box.

Another method to identify candidate roles would be to conduct a review of the literature to find underrepresented roles highlighted by the community. An exploratory search retrieved suggestions to add roles such as legal support (Craig et al., 2019), community engagement (Canadian Academy of Health Sciences, 2017), education and training (Hosseini et al., 2022b), technical and editorial support (Matarese & Shashok, 2019), and expert library and information support (Holmes, 2018) to CRediT’s list of roles. Somewhat like the previous approach, a shortcoming here is that members of the community who might have opinions about underrepresented roles or those with experiences related to the shortcoming of CROTs may not always share these through formal publications.

Given the mentioned challenges in adopting each approach, ethical and inclusive extension of CROTs requires using a combination of all mentioned approaches to compile a comprehensive list of candidate roles.

### Deciding what roles to include in the standard lists

Regardless of the approach taken to compile a shortlist, decisions need to be made about which roles to include in CROTs’ standard lists. In making these decisions, besides engaging user communities, appointing expert groups to reflect on received feedback and clarify future directions is necessary. Developers of CRediT and TaDiRAH have engaged with interested researchers to finalize their list of roles (Allen et al., 2012; Borek et al., 2014; 2021) and have also formed management committees to coordinate required activities (CRediT working group and TaDiRAH Board). However, besides engaging with the community and forming expert groups to manage related activities, a framework is needed to analyse candidate roles and make consistent decisions about which ones to add to the standard list of roles. To the best of our knowledge, none of the major CROTs have shared details about the used criteria to add new roles to their standard list.

A new approach to analyse candidate roles and define conditions under which a role should be added to CROTs’ standard list of roles is described in Hosseini (2021). The ‘significance threshold test’ is a conceptual framework to distinguish between core and non-core research roles involving two conditions: indispensability and specificity. Following this framework, candidate roles that are both indispensable (i.e., so important that research objectives cannot be achieved without them) and specific (i.e., directly associated with the questions and content of research, and constructively affect the reliability, validity and the justification of the reported data, claims, results and conclusions) would be added to the standard list of vocabularies. Hosseini (2021) applies this framework to various candidate roles found in the literature, three of which are mentioned in the Data S1 to this article.

One challenge of using this framework is that it requires a sophisticated analysis of roles, which is subject to personal/disciplinary interpretations. For instance, a specific role may pass the test in one domain but fail to satisfy both conditions in another domain (so-called inconclusive roles). Hosseini (2021) suggests that excluding these roles from standard lists would exclude relevant contributions on the basis that they are not of general interest to all areas, thereby denying researchers who make these contributions to be explicitly acknowledged. Including them, on the other hand, would add roles to the standard list that do not apply to all disciplines, yet are explicitly mentioned in the list of roles. While frameworks like this should be challenged and revised by the community, using a transparent process with publicly available criteria for the inclusion of roles facilitates a steady and democratic development of CROTs.

### Integrating new roles into the existing list of roles

Upon choosing one (or more) role from the list of candidate roles, they should be integrated into the existing list of roles. Depending on the structure and design of CROTs, this process may have different requirements. For instance, creating a closed list of top-level roles or role categories and an open and extendable list of lower-level roles is one approach to extend the list of roles. This approach is used by TaDiRAH as described in Borek et al. (2016). Eight high-level research goals are defined in TaDiRAH (i.e., Capture, Creation, Enrichment, Analysis, Interpretation, Storage, Dissemination and Meta-activity). Each goal includes three to seven methods (e.g., Conversion, Data Recognition, Discovering, Gathering, Imaging, Recording and Transcription are subsumed under the goal of Capture). While goals and methods have closed lists, TaDiRAH offers two open (and thus extendable) lists for research objects (e.g., data, image and text) and research techniques (e.g., scanning, debugging and photography) to better support ‘the rapid evolution of new techniques, without requiring constant revision to the core TaDiRAH terms’ (Borek et al., 2016). This way, as new techniques evolve or when new terms are suggested by the community via working on scope notes in Wikidata, the TaDiRAH Board (consisting of ‘the original core team, new developers and other contributors’) could discuss and add them to the open list (Borek et al., 2021).

In taxonomies with a single list of roles (i.e., closed taxonomies) such as CRediT, this process might involve different complexities. For instance, if the new role is an altogether novel role that would extend the total number of roles (e.g., in the case of CRediT, a hypothetical 15th role), the taxonomy should release a new version that may (or may not) be adopted by different actors in the scholarly commons. In such a scenario, reliable information exchange and consistency, which are among the advantages of using a closed taxonomy could be jeopardized. If, however, the new role is only going to extend the scope of an existing role by means of enhancing its definition, then papers tagged prior to the revision would have used the role with a different meaning, which may cause confusion. Furthermore, usefulness of the existing list of roles and their compatibility with the new roles need to be reviewed.

Either way, upon developing a revised version of the taxonomy, all existing adopters would have to make decisions about whether to upgrade their list of roles or not. While CROTs’ developers could provide incentives for upgrading the list of roles, they need to provide recommendations for cases where adopters do not upgrade.

## APPLICATION OF CROTS TO VARIOUS OBJECT TYPES AND RESEARCH AREAS

To date, CROTs have been most widely incorporated into authorship bylines of peer-reviewed articles and conference proceedings workflows using the 14 CRediT roles. We argue that with this limited application, the academic community is missing a good chance to reap the full benefits of CROTs. Although peer-reviewed publication is a critical activity in scholarship, limiting the applicability of CROTs to this form of output does not sufficiently address (1) contributions in disciplines where peer-reviewed articles and conference proceedings are either not a primary output (e.g., visual arts), or, they are only one of many important outputs (e.g., engineering); (2) publications involving significant non-author contributions; (3) publications involving irregular/ad hoc contributions (e.g., maintenance of computer systems) or non-research contributions (e.g., building security). These three aspects are further elaborated below.

There is an increasing appreciation of the diverse array of artefacts created during scholarly activities across different disciplines (e.g., datasets, software, hardware, protocols, maps, audiovisual resources, creative works, images, preliminary models, sketches, flowcharts and materials to support training and outreach activities). Since the 14 CRediT roles are tailored for peer-reviewed publications, they cannot capture all contributions necessary to create these other artefacts. Some non-text research outputs might not be accompanied by a journal article, or they might not always have a DOI. Contributions involved in creating these items cannot be fully captured using CRediT. The DataCite Metadata Working Group (2021), offers 21 role types (‘contributorType’—which is being expanded to accommodate CRediT), as well as 28 different resource types (‘resourceTypeGeneral’) to broaden the scope of what is meant with output beyond writing for peer-reviewed publications. Even so, it is reasonable to argue that there will be future roles and object types that will need to be captured. This is being addressed by the institutional repository community in interesting ways. For example, Zenodo accommodates a wide range of object types and contributor roles, by leveraging DataCite contributor roles and supporting customization in resource types in the updated Zenodo software framework, InvenioRDM (InvenioRDM, 2022). Contextualizing contributions to more object types using CROTs would allow assessment and evaluation workflows to aggregate this information, thereby incentivizing the use of CROTs.

One motivation to use CROTs is the recognition of contribution types that are often underrepresented in scholarly publications. Due to a range of reasons (e.g., lack of involvement in the task of writing, which according to the ICMJE is a prerequisite for becoming an author), some describe these contributions in acknowledgement sections (Hosseini et al., 2022a). Besides the discrepancy between the value and visibility of authorship bylines versus acknowledgement sections, since contributions mentioned in the acknowledgement section are described with a mixture of standardized vocabulary and free-text descriptions, they are not readily reusable and hence, not captured by scholarly metrics indicators. Even when CROTs fully cover the spectrum of involved roles in a project, in the absence of standardized guidelines and best practice, contributions mentioned in acknowledgement sections are reported with inconsistent formatting and structures and with varying degrees of detail (Paul-Hus et al., 2017). Although we have no perfect solution for these challenges, we see two possible ways to circumvent them. First, to the extent that is possible using current CROTs, all non-author contributions should be added in a machine-readable way to the acknowledgement section and the article’s openly accessible metadata. This way, future tools could access this information and capture them. A second solution (that might only apply to some projects) consists of creating and depositing other research artefacts with a different author list to cite in the paper. This way, acknowledged contributors in the paper would be authors of these derivative outputs. A link between derivative output(s) and the paper facilitates harvesting metrics and contributions (e.g., a dataset linked to a paper would be published with *dataset* authors, some of whom might be among the paper authors, for an example see Sehara, Zimmer-Harwood, Larkum, and Sachdev (2021) for the paper and Sehara, Zimmer-Harwood, Colomb, et al. (2021) for the dataset).

Note that with the development of systems of attribution for non-text publications, the question of their interoperability emerges. If we want to harvest contributor information and ascribe meaning to it, we need ways to map roles across different terminologies. Furthermore, developing tools that allow researchers to reuse the contribution list of one research output for the publication of related artefacts seem necessary. The use of an ontology like CRO might be an interesting solution to both issues. CRO provides more granular roles that could be automatically translated into a more general terminology in another output. For instance, one could imagine that roles like ‘Data Manipulation’ and ‘Data Modelling’ in a dataset, could be transcribed as ‘Data curation’ in the related research paper.

Appropriate recognition of technical contributions (e.g., tasks carried out by ICT engineers or field-based tasks in earth sciences) is of increasing interest to the scholarly community (McLaren & Dent, 2021). However, generic CROTs such as CRediT (that are meant to be applicable to more than one research area) seem insufficient in capturing nuanced technical contributions in different disciplines. For instance, CRediT’s roles of investigation and software are somewhat ambiguously defined, and when applied to complex projects, they flatten a broad spectrum of technical contributions that (preferably) should not be grouped together (Katz, 2015; Matarese & Shashok, 2019). Since irregular/ad hoc technical contributions with a significant impact on research results (e.g., burning prairies in spring/fall, as required in plant biology and restoration projects) might require specific certificates or unique health and safety measures, accurate attribution of credit to these contributions would be a testimony to their significance for advancing science in different fields. However, flattening these contributions (e.g., under the role of investigation), provides an incomplete account of involved tasks, and has resulted in various new initiatives focused on capturing the range of technical contributions within a specific role or community. In the case of software and especially Open Source software, there have been different initiatives to list specific contributions beyond code writing. In particular, the use of https://allcontributors.org makes it easy to attribute and show multiple types of roles when the software is hosted on GitHub. Furthermore, the application of CROTs in research software is being debated for storing in metadata and for citation, among members of the Force11 software citation working group and the citation file format community. Likewise, the EnviDat consortium created its own data-oriented role list (https://www.wsl.ch/datacredit/), inspired by CRediT and independent of DataCite, using their own database and visualization. Another unique example is APICURON (https://apicuron.org/), specifically developed to credit and acknowledge the work of biocurators (Hatos et al., 2021).

## INTEGRATION AND EXTENSION OF CROTS INTO SCHOLARLY WORKFLOWS

Scholarly publishing practices have been slowly changing. The adoption of CROTs in authorship submission forms is a major achievement of recent years, although advocacy for it began over 20 years ago (Rennie et al., 1997). We are optimistic that the tipping point to adopt CROTs in other areas beyond peer-reviewed publications might be imminent as well. Like Glasgow University which employs CROTs in their promotion workflow (Casci & McCutcheon, 2019), other academic institutions and scholarly professional could benefit from a wider integration of CROTs in their workflows. For example, CROTs can help to contextualize faculty contributions to research, teaching and service in promotion and tenure processes. Since these processes often lack structured or standard templates for assessing contributions (Sugimoto & Larivière, 2018), developing templates that incorporate CROTs could improve current practices. That said, this might involve challenges in some contexts. For example, while some contributions are measurable (e.g., via teaching evaluations, scholarly publications, service roles, national and international awards), others are more challenging to quantify (e.g., project/programme management roles, biocuration of datasets, software maintenance or architecture development and contribution to data standards).

This is further elaborated in a study by the ScholCommLab, which analysed guidelines used for review, promotion and tenure process at 129 academic institutions in the United States and Canada (Alperin et al., 2019). This study reported an emphasis on traditional scholarly products (such as publishing manuscripts, citation metrics and giving presentations) and a lack of specific guidance on how to capture non-traditional research outputs. Furthermore, the Humane Metrics Initiative (HuMetricsHSS, https://humetricshss.org/) has aimed to address issues with capturing metrics to evaluate promotion and tenure. They hosted workshops and conducted interviews with different groups affiliated with the Big Ten Academic Alliance institutions to improve how scholarly work is recognized and rewarded, and highlighted various opportunities, for example, a call for increased recognition of emerging or under-recognized research approaches, and a need to better track efforts and labour to ensure equity (Agate et al., 2022).

The challenge of quantifying inputs can be addressed by capturing CROTs on a curriculum vitae (CV), as annotations on publications, or in a non-standard reporting category such as Other Scholarly Products. Further development of applications such as Tenzing (Holcombe et al., 2020) and integration of CROTs in institutional systems such as repositories could create workflows to document and auto-populate contributions in other systems (e.g., by entering a DOI). This will allow research teams to indicate contributions across the wide range of necessary materials and activities associated with a project (e.g., for clinical trials, this can include artefacts such as recruitment materials, consent documentation, protocols and compliance documents). Establishing CROTs’ metadata in institutional repository systems allows individuals and groups to better characterize and attribute research outputs and activities, providing a foundation for integrity and equity in assessments (Hosseini & Holmes, 2021).

## SUPPORT AND ADOPTION OF CROTS

Well-developed workflows are critical to the successful use and adoption of CROTs. These workflows must be driven by all user groups, informed by their needs, and accompanied by complementary structures, training and resources to support them.

### User groups and advocacy for CROTs

**FIGURE 2:**
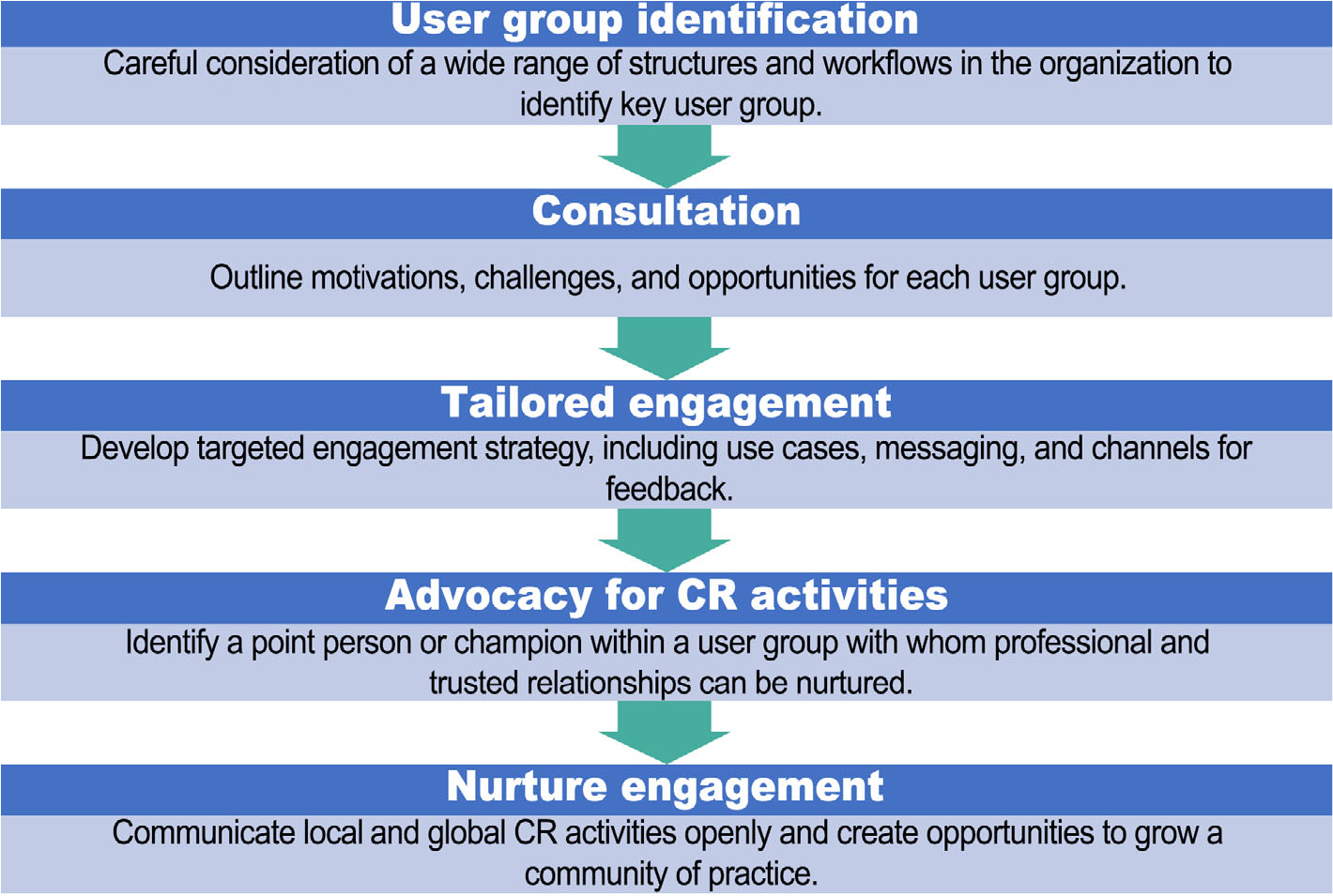
Strategies to encourage advocacy for contributor role ontologies and taxonomies.

To support CROTs’ integration throughout the research process, it is important to understand the wide range of user groups and their perspectives (e.g., universities and scientific institutions, funders, publishers, organizations that supply metrics and researchers; Hosseini & Holmes, 2021). User groups who directly benefit from integration of CROTs into workflows can serve as powerful partners, providing requirements, feedback and advocacy (Fig. 2).

Administrative and service units, faculty, data/system stewards and reporting infrastructure, each brings a unique perspective and motivation for integration of CROTs. However, since different cohorts within each user group might hold opposing views about how to reflect contributions using CROTs, effective consultation and tailored engagement are required to address their needs. For example, while research integrity officers and investigative bodies might be interested in knowing a highly detailed account of contributions to support their investigation of misconduct cases (e.g., Contributor X: Investigation [between April and June 2022, Validated by Contributor Y and Supervised by Contributor Z]; Writing the original draft [only the introduction section, all reviewed by Contributor Y]), department heads or tenure/promotion committees might prefer an overall account of contributions (e.g., Contributor X: Investigation; Writing the original draft).

### Use of CROTs prior to project completion

We believe that the incorporation of CROTs (whether CRediT or other schema) should not remain limited to a retrospective activity where a given work is annotated with CROTs after completion; CROTs may also be utilized when drafting research protocols, data management plans and funding proposals to articulate the required expertise and skillset based on projects’ goals and objectives. Using CROTs prior to projects’ commencement may in many cases provide only a rough estimation of what will ultimately occur in a project but is still useful as a planning document and for agreeing on involved roles within a project. Institutional Review Broads (IRBs) and ethics committees as well as funders could be among pressure points that can encourage groups to use CROTs already from the beginning of a project.

Some benefits of using CROTs from the initial stages of projects include:

- Funding agencies can assess different components of proposals beyond goals and deliverables, for example, based on contributors’ indicated skills, facilitating better evaluation informed by previous experiences with such tasks;
- Universities and scientific institutions can explore their staffs’/departments’ expertise, interdisciplinarity and the range of roles applied to their projects; Furthermore, with a shared understanding of CROTs, routine administrative and evaluative workflows such as reporting, or promotion and tenure can be supported and supplemented with more contextual perspectives;
- Researchers can reflect ongoing work, expertise and contributions on their CVs and faculty profiles in a more accurate and consistent manner; and
- Coordinators’ and supervisors’ team assembly can be improved, proactively leveraging expertise, rather than relying on personal relationships or serendipitous connections.

### Structures, training and resources

Dependable workflows are critical for the adoption of CROTs. However, embedding these workflows into institutions’ existing work ethic, culture and organizational structures are complicated and require a profound awareness of social and technical components of the academic research ecosystem. Researchers and lab groups, administrative and service units, data teams, technical system stewards and organizational leadership might benefit from CROTs’ integration. While these groups could use CROTs in different capacities to encourage accountability within their own context, a seamless integration of CROTs into institutional workflows requires a *nexus*, keeping all other user groups linked. We believe that libraries are perfectly positioned for this role.

Institutional libraries provide regular training and support for research, teaching and learning—and at the same time, are intricately connected with the publishing world and various research outputs. Like the support librarians provide around literature reviews, bibliographic management tools and training on topics such as copyright, citations and plagiarism, they can be institutional advocates and promoters of CROTs by:

- Liaising the concept of CROTs within their institutions and providing support for their integration into existing workflows;
- Facilitating conversations on campus with administrative units, data stewards and leaders;
- Providing training and resources to support uptake;
- Tracking and sharing online usability guides offered by publishers; and
- Enhancing accountability by providing support for reporting and compliance.

Current examples of libraries that have stepped up to support CROTs (e.g., TU/Delft, Simon Fraser University) show their potential for this purpose. These include activities such as development and presentation of information resources to leadership, academic or administrative units, targeted consultation services to support different user groups, usability guides to provide just-in-time support, and expert understanding of contributorship and how it can be presented in the broader information ecosystem.

## CONCLUSIONS

As CROTs become more widely accepted and implemented, the debate about extending their list of roles, application across disciplines and research object types, as well as their integration into diverse scholarly workflows gets more relevant and exponentially more complicated. In this article, we touched on various facets of this debate to inform the community about some of these complexities and discuss future opportunities. We acknowledge that what is discussed in this article is the tip of the iceberg, and there is much more to debate and explore about CROTs in general, and about each of the discussed items.

We predict that with the increased adoption of CROTs, some inconsistencies will ensue, but they can be harmonized and regulated at some level in the future. For the moment, inconsistencies are most noticeable at a journal level. For example, although many journals have adopted and made CRediT mandatory to use and show contributions in the published version of manuscripts (e.g., F1000), in others, using CRediT is either optional (e.g., Journal of Nicotine & Tobacco Research, see Munafò, 2020), or if mandatory, CRediT roles are not always shown in the published version (e.g., PLOS Biology, see Hilgetag & Zikopoulos, 2022). We see an opportunity for libraries to not only shepherd harmonization efforts, but also steward and support adoption at the discipline, institutional and researcher-levels.

Furthermore, we believe that community participation is essential for improvements in typologies to emerge and evolve. Through using community feedback and usage data, CROTs could review the usefulness of existing roles and identify new roles on an ongoing basis. Community-driven initiatives mentioned in this article are testimony to a growing appetite for an accurate attribution of credit and responsibilities within specific communities. Indeed, CROTs should belong and respond to community concerns and evolve as tools that support the varied contributions in modern scholarship. How to best approach and harmonize these efforts is undetermined though. For instance, many fields and subfields of science do not have a centralized governing council or democratically elected representatives to advocate for and solicit community views and feedback. Nevertheless, CROTs should remain open to revision and feedback to ensure that they are inclusive, relevant and can be further democratized by becoming available in a wide class of applications. Ultimately, future development of CROTs depends on advancements in technology and data, integration into routine scholarly workflows, and user-focused experience and support.

Since many journals have not yet adopted CROTs—likely due to the absence of a suitable or well-developed CROTs for their discipline or research object types (Allen et al., 2019), we recommend researchers to report underrepresented roles (e.g., those that are currently not among existing list of roles) in the acknowledgement sections of their publications. We also recommend publishers to share contributions under open access licences instead of only publishing them in acknowledgement sections that might be behind a paywall.

## Supplementary Material

Appendix S1

SUPPORTING INFORMATION

Additional supporting information may be found online in the Supporting Information section at the end of the article:

**Appendix S1** Supporting Information.

## Key points

- Contributor Role Ontologies and Taxonomies (CROTs) are more widely adopted but require refinement, further development and harmonization.
- Making decisions about what role(s) to add to CROTs’ existing list of roles is complicated but can be facilitated with consistent and transparent approaches.
- Application of CROTs is required outside traditional peer reviewed articles to ensure interoperability and maximize benefits of using CROTs in a range of research outputs.
- Different CROTs could be complementary rather than competitive, but better alignment requires coordination, guidelines, and infrastructure.
- Integration of CROTs within research workflows would benefit different user groups, and librarians may be best placed to advocate for such applications.
