# Supplementary material for: Evolution and adoption of contributor role ontologies and taxonomies
Source: Learn Publ. Author manuscript; Available in PMC 2026 Feb 27. (PMC12945348; doi:10.1002/leap.1496)
Supplement: Appendix S1 [file NIHMS2141973-supplement-Appendix_S1.docx]

## Evolution and Adoption of Contributor Role Ontologies and Taxonomies – Supplementary Document

When using the Significance Threshold Test to distinguish between core and non-core research roles, three verdicts might ensue, 1) roles that satisfy both indispensability and specificity conditions are deemed *significant*, 2) roles that do not satisfy both indispensability and specificity conditions are deemed *insignificant*, 3) roles whose indispensability and/or specificity cannot be sufficiently clarified are of *inconclusive* significance. In what follows, the application of the Significance Threshold Test on three roles that were identified by means of an exploratory search of the literature in Hosseini (2021) are directly quoted.

1. The role of *community engagement* is deemed **significant** because it satisfies both indispensability and specificity conditions:
2. Indispensability: “When research involving human subjects is conducted across cultural, structural or economic boundaries, community engagement efforts (e.g., interacting with the community via workshops and meetings) ensure that instrumental objectives of the research are achieved without flouting moral ideals or harming communities (Reynolds & Sariola 2018). These efforts aim to ensure that mandatory research policies and available best practices are adapted to fit the specific and local circumstances of each project (De Weger et al. 2018; Kaiser et al. 2016; Patten et al. 2019). Without these efforts, projects may be found to have trespassed research norms and be interrupted or suspended (Holzer et al. 2014)”.
3. Specificity: “Through facilitating the interaction between researchers and communities, this task directly affects “the well-being of the community” and their understanding of the research questions/content (Ahmed & Palermo 2010, p. 1383). This task enhances researchers’ understanding regarding power dynamics, leadership, social/political expectations, values and norms about the inhabitants of the place where research is conducted (Israel et al. 1998). Since findings in community-based research are sometimes inseparable from the relationship between the participants and inquirers, by providing cultural and historical input about the host community (Guba & Lincoln 1989), community engagement efforts constructively affect the reported data, claims, results and conclusions. For instance, providing knowledge about host community’s history/values may result in the development of solid data collection and data analysis techniques (Bracic 2018), both of which have a direct impact on the reported data, claims, results and conclusions.”

2) The role of *Communication and correspondence with other contributors and editorial teams* is deemed **insignificant** because it only satisfies the indispensability conditions:

1. Indispensability: “Communication and correspondence is a task that connects the contributors of a project to one another, and also to editorial teams. This task involves reporting individual contributions during the submission process, coordinating revision/resubmission efforts and responding to queries about published manuscripts (Kremenak 2010). Those conducting this task ensure that “all coauthors agree with the submitted and all subsequent revised versions of the manuscript” (Sterken 2011, p. 203). Accurate reporting of individual contributions and effective correspondence with editorial teams as well as project members require an overall awareness of individual contributions and ethical concerns such as conflicting interests (McNutt et al. 2018). Therefore, regardless of discipline or context, correspondence and communication is an indispensable task in the publication process that comes with distinct responsibilities (Ludvigsson et al. 2018).”
2. Specificity: “Although in contentious circumstance this task could become a non-routine task (e.g., in controversial situations such as authorship disputes, allegations of error/misconduct and retractions), since it only facilitates communication between parties, it does not make a direct and result-changing contribution to claims, data, results or conclusions. Even in cases where editors/peer-reviewers make suggestions that may affect content/conclusions, since corresponding contributors only communicate reviewers’ suggestions with the rest of the group, this task cannot be specific. Hence, this is an indispensable but generic task, and is deemed insignificant.^^[[1]](#footnote-1)^^”

3) The role of *legal support* is deemed **inconclusive** because its specificity cannot be sufficiently clarified:

1. Indispensability: “Legal support is sought in situations where patents, intellectual property rights or any financial interests are at stake. For example, in areas such as Biotechnology and Pharmaceutical research where both companies and universities make significant financial investments, the economic attraction/appeal of research ideas are dependent on their legal status, and necessitate seeking legal advice (Resnik 2007). Legal support is also sought when research results might have legal ramifications for a specific industry, e.g., exploring tobacco’s harmful effects (Crosbie & Glantz 2014), in projects that investigate participants who are involved in illegal activities or projects where vulnerable participants (e.g., children, subjects with disability) are recruited (Heesters et al. 2017).^[[2]](#footnote-2)^ In these cases, legal support is an indispensable task for achieving research objectives.”
2. Specificity: “Although many research projects require legal advice and support, this task is not always directly associated with research questions/content. Depending on whether legal support is provided in an advisory/administrative form or as part and parcel of a research endeavor, this task may or may not affect the status of data, claims, results and conclusions. For instance, in the field of digital systems where legal support is considered as a non-technical contribution and only relevant in keeping trade secrets (Reid & Rhodes 2016), it cannot affect research results and conclusions. Similarly, in Biomedical and Health contexts where legal support protects projects from legal disputes and fines that may result from illegal sharing of data (Fienberg 1994), it is not realistic for this task to have a direct impact on research results or conclusions.^^[[3]](#footnote-3)^^ On the contrary, in cases where legal support affects drug safety (Zweig 2017), or in public-scientist partnerships where legal advisors are actively present throughout the research process (e.g., Flint-Michigan water crisis); the provided legal advice constructively affects the reported data, claims and results (Soleri et al. 2016). In Biotechnology and Genetic Engineering where legal and technical expertise cannot be easily separated (because technical views need legal argumentation), legal support constructively affects research results and its conclusions (Godt 2016). Hence, since the specificity of this task is dependent on its scope/purpose, it cannot be declared as a clear-cut significant task, and is deemed inconclusive.”

## References

Ahmed, S. M., & Palermo, A.-G. S. (2010). Community Engagement in Research: Frameworks for Education and Peer Review. *American Journal of Public Health*, *100*(8), 1380–1387. <https://doi.org/10.2105/AJPH.2009.178137>

Bracic, A. (2018). For Better Science: The Benefits of Community Engagement in Research. *American Political Science Association*, *51*(03), 550–553. <https://doi.org/10.1017/S1049096518000446>

Crosbie, E., & Glantz, S. A. (2014). Tobacco industry argues domestic trademark laws and international treaties preclude cigarette health warning labels, despite consistent legal advice that the argument is invalid. *Tobacco Control*, *23*(3), e7–e7. <https://doi.org/10.1136/tobaccocontrol-2012-050569>

De Weger, E., Van Vooren, N., Luijkx, K. G., Baan, C. A., & Drewes, H. W. (2018). Achieving successful community engagement: A rapid realist review. *BMC Health Services Research*, *18*(1), 285. <https://doi.org/10.1186/s12913-018-3090-1>

Douglas, T. M. (2007). Ethics committees and the legality of research. Journal of Medical Ethics, 33(12), 732–736. <https://doi.org/10.1136/jme.2007.020479>

Fienberg, S. E. (1994). Sharing Statistical Data in the Biomedical and Health Sciences: Ethical, Institutional, Legal, and Professional Dimensions. *Annual Review of Public Health*, *15*(1), 1–18. <https://doi.org/10.1146/annurev.pu.15.050194.000245>

Godt, C. (2016). Experts and Politics in Patent Policy: The Final Report of the Expert Group on the Development and Implications of Patent Law in the Field of Biotechnology and Genetic Engineering of the European Commission, 17 May 2016. *IIC - International Review of Intellectual Property and Competition Law*, *47*(8), 960–980. <https://doi.org/10.1007/s40319-016-0529-y>

Guba, E. G., & Lincoln, Y. S. (1989). *Fourth Generation Evaluation*. SAGE.

Heesters, A. M., Buchman, D. Z., Anstey, K. W., Bell, J. A. H., Russell, B. J., & Wright, L. (2017). Power of Attorney for Research: The Need for a Clear Legal Mechanism. *Public Health Ethics*, *10*(1), 100–104. <https://doi.org/10.1093/phe/phw035>

Holzer, J. K., Ellis, L., & Merritt, M. W. (2014). Why We Need Community Engagement in Medical Research. *Journal of Investigative Medicine: The Official Publication of the American Federation for Clinical Research*, *62*(6), 851–855. <https://doi.org/10.1097/JIM.0000000000000097>

Israel, B. A., Schulz, A. J., Parker, E. A., & Becker, A. B. (1998). Review Of Community-Based Research: Assessing Partnership Approaches to Improve Public Health. *Annual Review of Public Health*, *19*(1), 173–202. <https://doi.org/10.1146/annurev.publhealth.19.1.173>

Kaiser, B. L., Thomas, G. R., & Bowers, B. J. (2016). A Case Study of Engaging Hard-to-Reach Participants in the Research Process: Community Advisors on Research Design and Strategies (CARDS)®. *Research in Nursing & Health*, *40*(1), 70–79. <https://doi.org/10.1002/nur.21753>

Kremenak, N. (2010). Authorship of the Research Report: Perils and Responsibilities. *Journal of Prosthodontics*, *19*(5), 420–421. <https://doi.org/10.1111/j.1532-849X.2010.00618.x>

Lakeman, R., & FitzGerald, M. (2009). The Ethics of Suicide Research. Crisis, 30(1), 13–19. <https://doi.org/10.1027/0227-5910.30.1.13>

Ludvigsson, J., Lefebvre, P., & Nerup, J. (2018). It is time to restore Rules for Authorship of scientific publications. *Pediatric Diabetes*, *19*(3), 586–586. <https://doi.org/10.1111/pedi.12572>

McNutt, M. K., Bradford, M., Drazen, J. M., Hanson, B., Howard, B., Jamieson, K. H., Kiermer, V., Marcus, E., Pope, B. K., Schekman, R., Swaminathan, S., Stang, P. J., & Verma, I. M. (2018). Transparency in authors’ contributions and responsibilities to promote integrity in scientific publication. Proceedings of the National Academy of Sciences, 115(11), 2557–2560. <https://doi.org/10.1073/pnas.1715374115>

Patten, C. A., Albertie, M. L., Chamie, C. A., Brockman, T. A., Gorfine, M., Nicholas, R., Bock, M. J., Okamoto, J. M., Penheiter, S. G., & Balls-Berry, J. E. (2019). Addressing community health needs through community engagement research advisory boards. *Journal of Clinical and Translational Science*, *3*(2–3), 125–128. <https://doi.org/10.1017/cts.2019.366>

Reid, J. B., & Rhodes, D. H. (2016). *Digital System Models: An investigation of the non-technical challenges and research needs*. 10.

Resnik, D. (2007). *The price of truth, how money affects the norms of science*. Oxford university press.

Reynolds, L., & Sariola, S. (2018). The ethics and politics of community engagement in global health research. *Critical Public Health*, *28*(3), 257–268. <https://doi.org/10.1080/09581596.2018.1449598>

Soleri, D., Long, J. W., Ramirez-Andreotta, M. D., Eitemiller, R., & Pandyaǁ, R. (2016). Finding pathways to more equitable and meaningful public-scientist partnerships. Citizen Science: Theory and Practice. 1(1): 9, 1(1), Article 1. <https://doi.org/10.5334/cstp.46>

Sterken, C. (2011). Writing a Scientific Paper III. Ethical Aspects. *EAS Publications Series*, *50*, 173–282. <https://doi.org/10.1051/eas/1150003>

Zweig, S. J. (2017). Selected Developments in Biotechnology Law and the Biotechnology Industry. *Biotechnology Law Report*, *36*(6), 269–271. <https://doi.org/10.1089/blr.2017.29048.sjz>

1. Some journals do not send their decision about a manuscript to all authors and only contact the corresponding author. Although in these cases too, the corresponding author is assumed to naturally be interested in sharing the decision and detailed comments with all the other co-authors. Partial disclosure of correspondence with journals with other authors could be seen as a questionable practice and abuse of both the editorial team’s and co-authors’ trust. [↑](#footnote-ref-1)
2. Furthermore, the importance of legal support is also shown by Resnik’s principle of Legality. This principle mandates scientists to follow laws pertaining to their work (Resnik 2005). [↑](#footnote-ref-2)
3. In its advisory/administrative form, this task is often conducted by non-core members such as research ethics committee members (some of whom should not be specifically recognised to keep their identity anonymous) (Douglas 2007; Lakeman & FitzGerald 2009), or universities’/laboratories’ technology transfer offices (Resnik 2007). Both of these entities operate independent of research projects and only provide administrative/supportive advice. [↑](#footnote-ref-3)
